# Effect of Obstructive Sleep Apnea Treatment on Lipids in Obese Children

**DOI:** 10.3390/children4060044

**Published:** 2017-06-01

**Authors:** Zarlasht Amini, Suresh Kotagal, Christine Lohse, Robin Lloyd, Swetha Sriram, Seema Kumar

**Affiliations:** 1 Department of Endocrinology, Children’s Hospital of Eastern Ontario, Ottawa, ON K1H 8L1, Canada; zamini@cheo.on.ca; 2 Division of Sleep Medicine; Mayo Clinic College of Medicine, Rochester, MN 55905, USA; kotagal.suresh@mayo.edu (S.K.); Lloyd.Robin@mayo.edu (R.L.); 3 Division of Biomedical Statistics and Informatics; Mayo Clinic College of Medicine, Rochester, MN 55905, USA; lohse.christine@mayo.edu; 4 Division of Pediatric Endocrinology and Metabolism, Mayo Clinic College of Medicine, Rochester, MN 55905, USA; swethasriram1990@gmail.com

**Keywords:** obstructive sleep apnea, childhood obesity, dyslipidemia, cholesterol, continuous positive airway pressure

## Abstract

Obesity in children is associated with several co-morbidities including dyslipidemia. Obstructive sleep apnea (OSA) is commonly seen in obese children. In adults, diagnosis of OSA independent of obesity is associated with cardiometabolic risk factors including dyslipidemia. There is limited data on the impact of treatment of OSA on lipids in children. The objective of the study was to examine the impact of treatment of OSA on lipids in 24 obese children. **Methods:** Seventeen children were treated with continuous positive airway pressure (CPAP) and five underwent adenotonsillectomy. Mean apnea hypopnea index prior to treatment was 13.0 + 12.1 and mean body mass index (BMI) was 38.0 + 10.6 kg/m^2^. **Results:** Treatment of OSA was associated with improvement in total cholesterol (mean change = −11 mg/dL, *p* < 0.001), and low-density lipoprotein cholesterol (mean change = –8.8 mg/dL, *p* = 0.021). **Conclusion:** Obese children should be routinely screened for OSA, as treatment of OSA favorably influences lipids and therefore decreases their cardiovascular risk.

## 1. Introduction

The prevalence of childhood and adult obesity in children has increased markedly in the last three decades. Currently, 17% of children in the United States are obese [1]. Obesity is associated with several cardiometabolic risk factors, including dyslipidemia [2,3,4]. Obesity is also a risk factor for obstructive sleep apnea (OSA) and, consequently, the prevalence of OSA in obese children is higher than in non-obese children [5,6,7]. In a population-based study, the risk of OSA was increased four- to five-fold in obese children [8].

In adults, diagnosis of OSA independent of obesity is associated with cardiometabolic risk factors including dyslipidemia [9]. It is not clear, however, if OSA in children is also associated with metabolic dysfunction independent of obesity [10,11]. There are still inconsistencies in the data on the impact of treatment of OSA on lipid levels in children [12,13,14,15]. Most of these studies have examined the impact of adenotonsillectomy on lipids in normal-weight and obese children [12,13,14]. However, the effect of positive airway pressure (PAP) on lipid levels in overweight and obese children with OSA remains unknown. Therefore, the objective of our study was to examine the effect of treatment of OSA on lipids in overweight and obese children and adolescents.

## 2. Materials/Methods

### 2.1. Subjects

This retrospective study was approved by the Mayo Clinic Institutional Review Board. After verification of research authorization under Minnesota statute 144.335, medical records of subjects aged 2 to 18 years, who had undergone evaluation at the Center for Sleep Medicine and Pediatric Endocrine Clinic at Mayo Clinic, Rochester, MN between 1 January 2000 and 31 December 2010, were reviewed. 

Medical records of patients were included if they had a body mass index (BMI) greater than or equal to the 85th percentile for age and gender and apnea–hypopnea index (AHI) of ≥1 per hour on overnight polysomnography (PSG) for evaluation of OSA [16]. Those patients who had measurement of fasting lipids before and after treatment of OSA were selected for further review. Subjects were excluded if they had predominant central sleep apnea, type 1 or type 2 diabetes mellitus, craniofacial malformation, neuromuscular disorders, genetic syndromes, cancer, chronic inflammatory disorder or if they were using systemic steroids, lipid lowering medications, oral hypoglycemics, metformin or insulin.

### 2.2. Initial Assessment

Demographic data were collected for patients, including age, sex, self-declared race/ethnicity, height and weight. Age- and sex-specific BMI percentiles were determined with the 2000 Center for Disease Control (CDC) growth charts [16]. In addition, the BMI *z*-scores of the subjects were determined, using the age-specific and sex-specific median BMI (M), generalized coefficient of variation (S), and the power of the Box–Cox transformation (L) with this formula:

Z = (X/M) L − 1/(LS), for L ≠ 0 and Z = ln (X/M)/S, for L = 0

where X is the individual patient’s BMI.

### 2.3. Polysomnography

All subjects underwent PSG in accordance with standards defined by the American Academy of Sleep Medicine (AASM). For subjects undergoing nocturnal PSG prior to 2007, the Rechtschaffen and Kales sleep scoring criteria were used [17], whereas for subjects enrolled after 2007, the AASM scoring criteria were applied [18]. The following signals were included: EEG (leads C3-A2, C4-A1, O3-A2, and O4-A1), submental and leg electromyogram. ECG and heart rate were recorded simultaneously. Snoring, oronasal air flow and nasal pressure and end tidal carbon dioxide were also recorded. Thoracic and abdominal respiratory effort was monitored using respiratory inductance plethysmography. In accordance with the pediatric respiratory scoring rules established by AASM, apnea was defined as a 90% decrease in signal amplitude of the oronasal thermocouple for at least 2 breaths; hypopnea was defined as a greater than or equal to 50% fall in amplitude of the nasal pressure transducer for at least 2 breaths and was associated with an arousal, or at least 3% oxygen desaturation. AHI was defined as the total number of apneas and hypopneas occurring per hour of sleep. Obstructive AHI (O-AHI) was also noted. It is known that children with sleep-related upper airway obstruction may show increased respiratory event-related arousals rather than frank apneas [19]. Consequently, we also obtained the total arousal index and the percentage of arousals that were related to respiratory events.

### 2.4. Biochemical Analyses

Fasting total cholesterol, high-density lipoprotein (HDL) cholesterol and triglyceride levels were measured by an enzymatic colorimetric assay (Roche Diagnostics, Indianapolis, IN, USA). Low-density lipoprotein (LDL) cholesterol was calculated using the Friedewald equation [20].

Calculated LDL cholesterol = Total Cholesterol − HDL cholesterol − (Triglycerides ÷ 5)

Fasting glucose was measured by the photometric hexokinase method (Roche Diagnostics). Baseline biochemical studies were obtained at the time of pediatric endocrinology consultation, prior to treatment of OSA. Follow-up lipid screens were obtained at subsequent endocrinology follow-up clinic visits. Elevated total cholesterol was defined as >200 mg/dL, elevated LDL cholesterol as ≥130 mg/dL, low HDL as <40 mg/dL and elevated triglycerides as ≥100 mg/dL for ages 0–9 years and ≥130 mg/dL for ages 10–19 years.

### 2.5. Statistical Analysis

Continuous variables were summarized with means, standard deviations (*SD*), medians, and ranges, while categorical features were summarized with frequency counts and percentages. Associations among variables was evaluated using Spearman rank correlation coefficients and Kruskal–Wallis, Wilcoxon rank sum, chi-square, and Fisher exact tests, as appropriate. Changes in features from baseline to most recent follow-up within the same child were evaluated using paired *t*-test and Wilcoxon signed-rank tests. Analyses were performed using the SAS software package (Version 9.0; SAS Institute Inc., Cary, NC, USA) and *p*-values < 0.05 were considered statistically significant.

## 3. Results

Twenty-four children had lipid levels at baseline and following treatment of OSA. Out of these, 18 children had a repeat fasting glucose drawn at both baseline and following treatment of OSA. The mean age at the time of the initial diagnostic study was 11.8 ± 3.4 years (median = 12; range = 5–16), 54% of the subjects were males (Table 1). Eighteen of 24 patients were Caucasian, 2 were African-American, 1 Hispanic, 1 Middle Eastern and 2 patients had no specified race. The mean BMI for the study population was 38.0 ± 10.6 kg/m^2^ (median = 38; range = 23–62). The mean AHI was 13.0 ± 12.1 (median = 11; range = 1–48). The median O-AHI was 1 (mean = 1.5, *SD* = 2.3, range = 0–9). Median total arousal index was 12 (mean = 17.3, *SD* = 11.9, range = 5–43) and median percentage of respiratory event-related arousals was 44.5% (mean = 46.6, *SD* = 25, range = 11–95).

Six out of 24 (25%) children had elevated total cholesterol, 4/22 (18.1%) had elevated LDL cholesterol, 10/22 (45.5%) had low HDL cholesterol and 12/24 (50%) had elevated triglycerides. At baseline, there was no correlation between OSA severity and fasting glucose, total cholesterol, HDL cholesterol, triglycerides and calculated LDL cholesterol (*p* > 0.05).

The follow-up studies were drawn at a mean of 19 months (median 12.5; range 3–46) following initiation of treatment of OSA. Of 24 children who had baseline and follow-up lipids, 17 were treated with continuous positive airway pressure (CPAP) and five underwent adenotonsillectomy. One patient was treated with bilevel positive airway pressure device (BPAP) and one child with an AHI of 1 was treated with a steroid nasal spray (Fluticasone). Median nadir oxygen saturation was 89% (range 51–94). Overnight PSG was performed in five patients after treatment of OSA (three following positive airway pressure and two following adenotonsillectomy). The AHI in these patients decreased from 10.8 + 3.7 to 3.66 + 2. The titration of pressure settings was performed in in-lab polysomnogram in all three patients treated with PAP (2 CPAP, 1 BPAP). The remaining CPAP patients were treated with auto-adjusting CPAP.

When all 24 patients were considered as a group together, mean total serum cholesterol decreased from 179.8 mg/dL to 168.9 mg/dL (mean change = −11, *p* < 0.001; Table 2) and mean LDL cholesterol decreased from 111.7 mg/dL to 103 mg/dL (mean change = –8.8 mg/dL, *p* = 0.021; Table 2). There was no significant change in HDL cholesterol, triglycerides or fasting glucose (*p* > 0.05 for all; Table 2).

We then performed subgroup analysis by dividing patients into two categories: those receiving CPAP/BPAP (*n* = 18) and those having undergoing adenotonsillectomy or receiving fluticasone (*n* = 6). There was a significant decrease in total cholesterol and LDL cholesterol in the 18 subjects treated with CPAP/BPAP (mean change = −8.1 mg/dL, *p* = 0.018 and −8.6 mg/dL, *p* = 0.038 respectively; Table 3). Total cholesterol also decreased in the subgroup that underwent adenotonsillectomy or received fluticasone (mean change = −19.5 mg/dL, *p* = 0.02). However, there was no significant change in LDL cholesterol in that subgroup (*p* > 0.05). Additionally, there was no significant change in HDL cholesterol, triglycerides or fasting glucose in either of the subgroups, and BMI did not significantly change during the follow-up either (*p* = 0.32).

## 4. Discussion

In the current study, we found that treatment of OSA in obese children was associated with improvement in total cholesterol and LDL cholesterol, despite no change in their weight status. To our knowledge, this is the first longitudinal study to examine the impact of treatment of OSA with CPAP or BPAP on lipids in overweight and obese children who are at an increased risk for dyslipidemia.

The findings of improvement in total cholesterol and LDL cholesterol that we noted in our study have clinical relevance, given the evidence for the continuous, graded relationship between the total plasma cholesterol concentration and coronary heart disease events and mortality [21,22]. We recognize that the OSA in our patients was in the mild-to-moderate range, and is reflected mainly in increased respiratory event-related arousals. Keeping this in mind, the degree of improvement in the lipid profile is still impressive. To our knowledge, the only other previous study that examined the impact of treatment of OSA with CPAP in 11 obese children did not report on changes in lipids [15]. The findings in our study are in agreement with those in a previous study in obese children by Gozal et al., who reported significant improvement in not only total cholesterol and LDL cholesterol, but also HDL cholesterol and triglycerides following adenotonsillectomy [12]. Adenotonsillectomy was also noted to be associated with improvement in total cholesterol (mean change = 3.9 mg/dL) in another study, although the number of obese children in that particular study was too small to draw definite conclusions [23].

Our findings of improvement in total cholesterol and LDL cholesterol in obese children following treatment of their OSA contrast with two other studies [13,14]. In a cohort of 69 children (1/3 of whom were obese), Koren et al. noted improvement in HDL cholesterol but not in total cholesterol and LDL cholesterol, following tonsillectomy and adenoidectomy [13]. Additionally, in a multicenter “The Childhood Adenotonsillectomy Trial (CHAT)”, in which children were randomized to either early adenotonsillectomy or watchful waiting with supportive care, over a 7-month follow-up period following adenotonsillectomy, no improvement in lipids was also noted [14]. However, these inconsistencies are likely related to significant differences in characteristics of the study subjects and duration of follow-up. The mean age of the children in our study was older (11.8 years in our study, 7 years and 6.3 years in the CHAT trial and study by Koren et al., respectively) as well as heavier (mean BMI *z*-score = 2.6 in our study, 0.9 and 1.43 in the CHAT trial and study by Koren et al., respectively) [13,14]. Additionally, as expected due to higher BMI, the cardiometabolic profile was less healthy in our study, with children having higher total cholesterol, higher LDL cholesterol, higher triglycerides and lower HDL cholesterol compared to subjects in the other two studies [13,14]. These differences are also likely related to differences between the treatment modalities for OSA, with the majority of our patients receiving CPAP, whereas all subjects in the other two studies underwent adenotonsillectomy [13,14].

Our findings of no change in fasting glucose are similar to those reported in two previous studies with obese children, one following adenotonsillectomy [12] and the other one following treatment with CPAP [15].

The mechanisms by which OSA and lipids are linked may be mediated by the effect of chronic intermittent hypoxia on lipid biosynthesis and lipid peroxidation [24,25,26]. Chronic intermittent hypoxia in mice has been shown to result in upregulation of the control of multiple genes: (1) cholesterol and fatty acid biosynthesis (malic enzyme and acetyl coenzyme A (CoA) synthetase); (2) predominantly fatty acid biosynthesis (acetyl-CoA carboxylase and stearoyl-CoA desaturases 1 and 2); and (3) triglyceride and phospholipid biosynthesis (mitochondrial glycerol-3-phosphate acyltransferase) [24,25,26]. In a murine model of intermittent hypoxia, during sleep that mimicked OSA in lean and obese mice, substantial increases in total and LDL cholesterol were noted in conjunction with a 1.5- to 2-fold increase in lipoprotein secretion, and upregulation of hepatic stearoyl coenzyme A desaturase 1, a critical enzyme of lipid biosynthesis [17,18,24,25,26]. Additionally, severe chronic intermittent hypoxia in mice led to markedly increased lipid peroxidation in the liver [18,24,25,26].

Our study has many strengths, including the inclusion of obese children who are most at risk for cardiometabolic risk factors (i.e., dyslipidemia) and the use of CPAP in the majority of study subjects as a treatment modality, as the impact of CPAP on lipids in obese children with OSA has not been previously studied. Another strength is the weight stability of patients during the follow-up period, thereby ruling out an impact of change in weight status on lipid profile.

Certain limitations did arise within the study, which should be addressed in future follow-up research within this field. First, our study had a lack of follow-up PSG in the majority of study participants and a lack of adherence data after CPAP. These challenges have been encountered in previous studies as well and continue to make it difficult to study the direct impact of CPAP on cardiometabolic risk factors in children with OSA [15]. Other limitations included the small sample size and heterogeneity of the study population, both in terms of age and pubertal stage, as lipid levels change with puberty and therefore, the lack of data on pubertal progression during the duration of the study was a limitation. Additionally, although the severity of obesity did not change, we did not have information on dietary habits and physical activity, both of which can impact lipids. Finally, the findings cannot be generalized to all ethnic groups, as the study population was predominantly white.

In conclusion, this study shows that treatment of OSA in overweight and obese children and adolescents is associated with improvement in total cholesterol and LDL cholesterol levels. Further studies are warranted to assess the impact of treatment of OSA on lipids and other cardiovascular risk factors in obese children. Consequently, obese children undergoing evaluation in primary care should be routinely screened for OSA, as treatment of OSA may be an additional strategy to decrease their cardiometabolic risk.

## Figures and Tables

**Table 1:** Anthropometric and laboratory characteristics of study subjects.

Variable	Mean ± *SD* (Median; Range)
Age (years)	11.8 ± 3.4 (12; 5–16)
Male, *n (%)*	13 (54)
Female *n* (/%)	11 (46)
BMI (kg/m^2^)	38.0 ± 10.6 (38; 23–62)
BMI percentile	98.9 ± 1.8 (99.6; 92.1–100)
BMI *z*-score	2.6 ± 0.5 (2.7; 1.4–3.5)
Fasting glucose (mg/dL) ^a^	92.3 ± 9.4 (93; 67–110)
Total cholesterol (mg/dL) ^b^	179.8 ± 30.7 (178; 128–227)
LDL cholesterol (mg/dL) ^c^	109.9 ± 24.3 (111; 69–157)
HDL cholesterol (mg/dL) ^c^	42.8 ± 9.8 (41; 25–68)
Triglycerides (mg/dL) ^b^	130.7 ± 79.4 (116; 47–430)
AHI	13.0 ± 12.1 (11; 1–48)
Mild OSA, (AHI 1–4), *n* (%)	4 (17)
Moderate OSA (AHI 5–10), *n* (%)	8 (33)
Severe OSA (AHI > 10), *n* (%)	12 (50)
Total Arousal Index	17.3 ± 11.9 (12, 5–43)
Respiratory event related arousals (%)	46.6 ± 25 (44.5, 11–95)
CPAP, *n* (%)	17 (71)
Adenotonsillectomy, *n* (%)	5 (21)
BPAP, *n* (%)	1 (4)
Flonase, *n* (%)	1 (4)
Total Recording Time (minutes)	326 ± 137.2 (266; 155–539)
Total Sleep Time (minutes)	283.1 ± 122.4 (241.5; 124.5–487)
Sleep Efficiency (%)	87 ± 9 (89.6; 67.4–98.1)

^a^ *n* = 18; ^b^ *n* = 24; ^c^ *n* = 22; BMI = body mass index; LDL = low-density lipoprotein; HDL = high-density lipoprotein; AHI = apnea–hypopnea index; OSA = obstructive sleep apnea; CPAP = continuous positive airway pressure; BPAP = bilevel positive airway pressure device.

**Table 2:** Change in laboratory parameters from baseline.

Laboratory Parameter *	Baseline **	Post-Intervention **	Change **	*p*-Value
Total Cholesterol ^a^	179.8 (177.5; 128–227)	168.9 (170.5; 104–225)	−11.0 (−12.5; −37 to 15)	<0.001
LDL Cholesterol ^b^	111.7 (111; 72–157)	103.0 (105; 37–146)	−8.8 (−4.5; −41 to 21)	0.021
HDL Cholesterol ^b^	43.2 (41.5; 25–68)	42.7 (41; 17–73)	−0.5 (−3; −15 to 31)	0.81
Triglycerides ^a^	130.7 (115.5; 47–430)	141.7 (130; 47–138)	11 (14; −277 to 171)	0.54
Fasting glucose ^c^	92.2 (93; 67–100)	90.9 (68–118)	−1.3 (−1.5; −16 to 27)	0.63

* mg/dL; ** Data are represented as mean (median; range); ^a^ *n* = 24; ^b^ *n* = 22; ^c^ *n* = 18.

**Table 3:** Change in laboratory parameters from baseline in those treated with CPAP/BPAP.

Laboratory Parameter *	Baseline **	Post-Intervention **	Change **	*p*-Value
Total Cholesterol ^a^	175 (171.5; 128–227)	166.9 (171.5; 104–225)	−8.1 (−12.2; −27 to 15)	0.018
LDL Cholesterol ^b^	106.2 (110; 72–138)	97.6 (99; 37–144)	−8.6 (−7.5; −41 to 21)	0.038

* mg/dL; ** Data are represented as mean (median; range); ^a^ *n* = 18; ^b^ *n* = 17.
